# Anterior Segment Optical Coherence Tomography Imaging of Filtering Blebs after Deep Sclerectomy with Esnoper-Clip Implant: One-year Follow-up

**DOI:** 10.5005/jp-journals-10008-1169

**Published:** 2015-01-15

**Authors:** Luisa Vieira, Manuel Noronha, Vanessa Lemos, Maria Reina, Teresa Gomes

**Affiliations:** Resident, Department of Ophthalmology, Central Lisbon Hospital Center Lisbon, Portugal; Resident, Department of Ophthalmology, Central Lisbon Hospital Center Lisbon, Portugal; Resident, Department of Ophthalmology, Central Lisbon Hospital Center Lisbon, Portugal; Consultant, Department of Ophthalmology, Central Lisbon Hospital Center Lisbon, Portugal; Consultant, Department of Ophthalmology, Central Lisbon Hospital Center Lisbon, Portugal

**Keywords:** Deep sclerectomy, Intraocular pressure, Esnoper-Clip implant, AS-OCT.

## Abstract

**Purpose:** To describe the technique of deep sclerectomy with the new Esnoper-Clip® implant, the clinical outcome and the anatomic characteristics of filtering blebs, using anterior segment optical coherence tomography (AS-OCT).

**Methods:** A prospective case-series study was conducted in five eyes (5 patients) with open angle glaucoma. The fornix-based deep sclerectomy with Esnoper-Clip® implant was done by the same surgeon. In one case, mitomycin C was used during surgery. All participants underwent a complete ophthalmic examination and AS-OCT (Visante®) preoperatively, then at each follow-up visit, at 1 day, 1 week, 1 month, 6 months and 1 year postoperatively. Scans were obtained through sagittal and transversal plans to the implant.

**Results:** Intraocular pressure (IOP) was significantly reduced (p < 0.05) from a mean preoperative value of 23.4 ± 8.6 mm Hg (n = 3.8 glaucoma medications) to a postoperative value of 6.0 ± 2.5 (n = 0), 10.6 ± 5.4 (n = 0), 13 ± 1.6 (n = 0.4), 12.4 ± 2.1 (n = 0.2) and 14.4 ± 1.5 (n = 0.2) at 1 day, 1 week, 1 month, 6 months and 1 year respectively. AS-OCT allowed the visualization of the two plates of the implant (scleral and suprasciliary), the trabeculodescemetic membrane and the hyporeflective spaces in the bleb wall thickness and in suprascleral and suprachoroidal localizations. An immediate postoperative hypotony and an anteriorization of the implant associated to trabeculodescemetic membrane rupture, were detected, although without significant clinical repercussions.

**Conclusion:** Our first five deep sclerectomy with Esnoper-Clip implantation analysis suggest an effective and well-tolerated method to reduce IOP. AS-OCT is a noninvasive imaging technique that allows the anatomic analysis of the drainage mechanisms after glaucoma surgery.

**How to cite this article:** Vieira L, Noronha M, Lemos V, Reina M, Gomes T. Anterior Segment Optical Coherence Tomography Imaging of Filtering Blebs after Deep Sclerectomy with Esnoper-Clip Implant: One-year Follow-up. J Curr Glaucoma Pract 2014;8(3):91-95.

## INTRODUCTION

In the last years, deep sclerectomy (DS) has gained importance in glaucoma surgical treatment. In contrast to trabeculectomy, it enhances aqueous humor outflow acting on the major resistance site without entering the anterior chamber, thus reducing the incidence of intra and postoperative overfiltration, hypotony and its potential sequelae.^1-8^ Moreover, the incidence of cataract,^8-10^ hyphema,^5^ endothelial damage^11^ and macular edema is lower^5^ with DS. Although it is widely accepted that the DS is safer, remains some controversy about the intraocular pressure (IOP)-lowering effects. Chiselita et al^12^ and Cheng et al^1^ described lower efficacy than trabeculectomy, in contrast to El Sayyad et al^13^ that described similar efficacy. Mitomycin-C and many types of implants (Aquaflow,® SK-Gel,® T-flux,® Esnoper®)^2^ have been used to improve the efficacy of this technique, with reported similar results to trabeculectomy,^4,5,14^ although it is not consensual.^6,8^ Esnoper-Clip® is a nonreabsorbable implant, which objective is to maximize all aqueous humor drainage pathways.

Anterior segment optical coherence tomography (AS-OCT) is a noninvasive, noncontact imaging technology that provides high-resolution cross-sectional images of anterior segment.^15^ It has been used to evaluate filtering blebs after trabeculectomy^16-19^ and DS,^16,17,20-23^ allowing a better understanding about aqueous humor drainage pathways and its function.

The purpose of this study was to describe the technique of deep sclerectomy with the new Esnoper-Clip^®^ implant, the clinical outcome (IOP decrease and complications with one year follow-up) and the anatomic characteristics of filtering blebs, using AS-OCT. To our knowledge, this is the first clinical study to evaluate this new implant.

## METHODS

### Participant Selection

A prospective case-series study was conducted in five eyes of five patients (4♂: 1♀), with a mean age of 61 ± 15.6 years, followed in Glaucoma Section of Ophthalmology Department of Central Lisbon Hospital Center (Table 1). Three patients had primary open-angle glaucoma (POAG) and two pseudoexfoliative glaucoma (PEXG), with characteristic structural and functional findings. Two of them had previous cataract surgery for more than 6 months. All patients had uncontrolled glaucoma despite being on maximal medical therapy.

**Table 1:** **Table 1:** Patients’ features

*Patient*		*Age*		*Gender*		*Eye*		*Type of* *glaucoma*		*Previous* *ocular* *surgeries*	
1		58		♂		Right		POAG		Cataract	
2		71		♀		Left		PEXG		No	
3		51		♂		Left		POAG		No	
4		43		♂		Right		PEXG		No	
5		82		♂		Left		POAG		Cataract	

## PROCEDURES

**Fig. 1:**
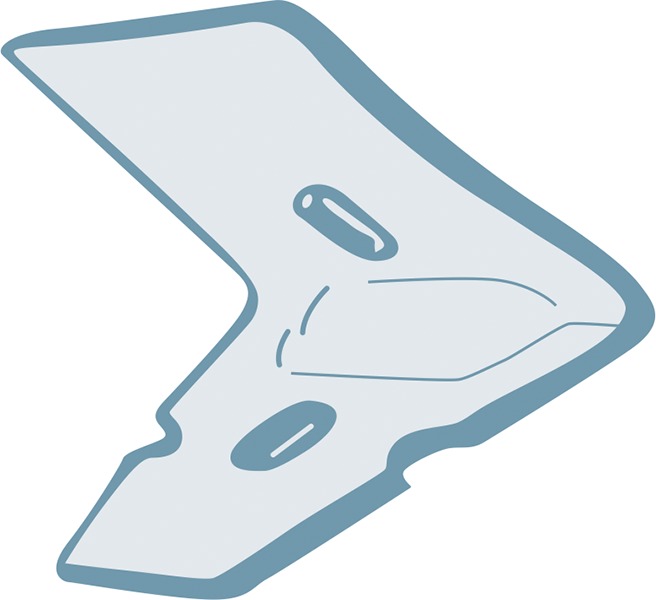
Esnoper-Clip® implant

Esnoper-Clip® is a nonabsorbable foldable implant with two plates (scleral and supraciliary), composed by 2-hydroxyethyl methacrylate, a nonionic polymer with low tendency for protein deposits. Its length, unfolded, is 5.5 mm (3 mm scleral plate and 2.5 mm supraciliary plate), the base width is 2.2 mm and the thickness is 0.2 mm (Fig. 1).

The fornix-based deep sclerectomy with Esnoper-Clip® implant was done by the same surgeon. After scleral exposure, a superficial flap of a 1/3 of sclera thickness was created (5.0 × 5.0 mm), 1 mm beyond the limbus. Then, a second deeper flap was done (4.0 × 4.0 mm), the peeling of Schlemm’s canal was completed and the deepest sclera flap was excised, leaving the trabeculodescemetic membrane patent. Esnoper-Clip^®^ implant was inserted, one plate in the intrascleral space and the other in the supraciliary space. At last, the scleral flap was sutured with monofilament 10-0 as long as the conjunctiva. In one case, mitomycin C was used during surgery (ethnic and inflammatory factors).

**Fig. 2:**
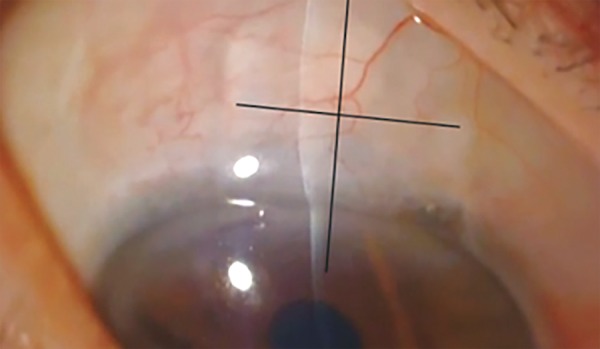
Anterior segment optical coherence tomography scans were done through sagittal and transversal plans to the implant (as shown by lines)

All participants underwent a full ophthalmologic examination (including measurement of IOP with Goldmann tonometer) and AS-OCT (Visante®) preope-ratively, then at 1 day, 1 week, 1 month, 6 months and 1 year postoperatively. Scans were done through sagittal and transversal plans to the implant (Fig. 2).

The number of glaucoma medications needed to lower IOP, after the surgical procedure, was documented.

## STATISTICAL ANALYSIS

Statistical analysis was conducted using SPSS Statistics version 20.0. The average and standard deviation (SD) of IOP and number of glaucoma medications were calculated. These values were compared between the different follow-up times using Mann-Whitney test. p-values less than 0.05 were considered statistically significant.

### Ethics Statement

The study was approved by the Human Research Ethics Committee of the Central Lisbon Hospital Center and followed the tenets of the Declaration of Helsinki. Written informed consent was obtained from all participants.

## RESULTS

Intraocular pressure and number of glaucoma medications were significantly reduced (p < 0.05) from a preoperative value of 23.4 ± 8.6 mm Hg (n = 3.8 glaucoma medications) to a postoperative value of 6.0 ± 2.5 (n = 0), 10.6 ± 5.4 (n = 0), 13 ± 1.6 (n = 0.4), 12.4 ± 2.1 (n = 0.2) and 14.4 ± 1.5 (n = 0.2) at 1 day, 1 week, 1 month, 6 months and 1 year respectively (Table 2).

**Table 2:** **Table 2:** Intraocular pressure and number of glaucoma medications (n) preoperatively and at 1 day, 1 week, 1 month, 6 months and 1 year postoperatively

		*Preiperatively*				*1 day*				*1 week*				*1 month*				*6 months*				*1 year*			
		*IOP*		*n*		*IOP*		*n*		*IOP*		*n*		*IOP*		*n*		*IOP*		*n*		*IOP*		*n*	
Average ±		23.4		3.8		6.0 ±		0 ±		10.6		0 ±		13 ±		0.4 ±		12.4 ±		0.2 ±		14.4 ±		0.2 ±	
standard		±		±		2.5		0		±		0		1.6		0.9		2.1		0.4		1.5		0.5	
deviation		8.6		0.4						5.4															

**Figs 3A to C:**
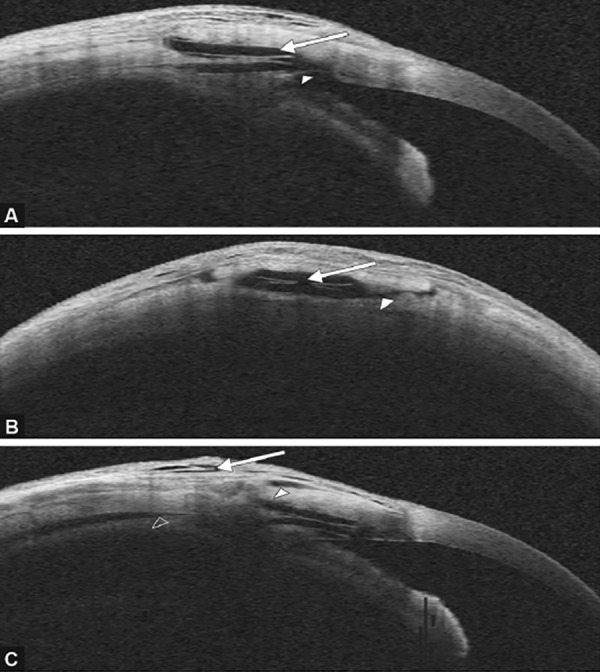
Anterior segment optical coherence tomography: (A) Longitudinal scan showing the double plate of Esnoper-Clip® implant (arrow) and an intact trabeculodescemetic membrane (arrow head), (B) transversal scan showing the double plate of the Esnoper-Clip® implant (arrow) and an hypore**a**ctive space suggestive of a scleral route (arrow head) between intrascleral space and suprascleral space, (C) longitudinal scan showing hypore**a**ctive spaces in the bleb wall thickness (arrow) and in suprascleral (arrow head) and suprachoroidal localizations (empty arrow head)

**Figs 4A and B:**
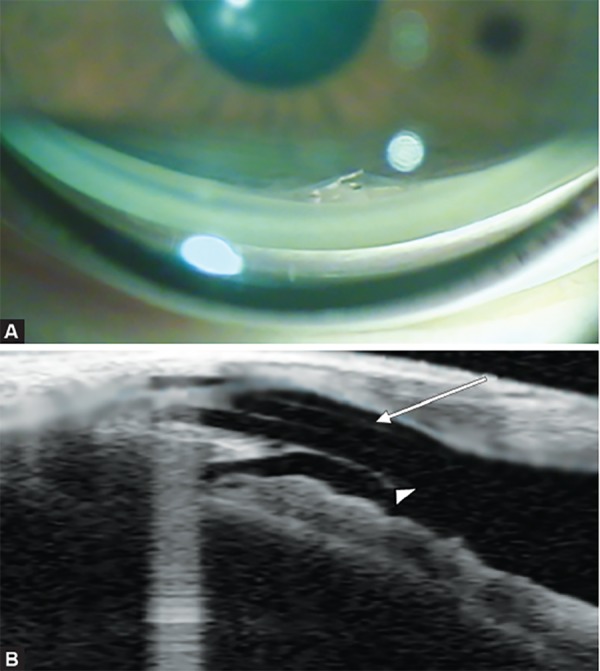
Anteriorization of the Esnoper-Clip® implant: (A) Image of the implant in the anterior chamber seen by gonioscopy, (B) longitudinal scan of AS-OCT showing the double plate of Esnoper-Clip^®^ implant (arrow) and the ruptured trabeculodescemetic membrane (arrow head)

Anterior segment optical coherence tomography allowed the visualization of the two plates of the implant (scleral and supraciliary), the trabeculodescemetic membrane and the hyporeflective spaces in the bleb wall thickness and in suprascleral and supra-choroidal localizations (Figs 3A to C).

As complications we have registered one eye with immediate postoperative hypotony, which was solved in 9 days, without sequelae. In other patient, we verified the anteriorization of the Esnoper-Clip^®^ implant associated to trabeculodescemetic membrane rupture at 6-month follow-up (Figs 4A and B).

## DISCUSSION

Previous studies suggested that DS with the use of implants have good efficacy and low rate of perioperative complications.^1-8,14^ During the first year of follow-up, we found a maintained postoperative IOP decrease after DS with Esnoper-Clip^®^ implant, similar to the values published with other implants,^20,22^ with almost no need of glaucoma medications. The hypotony reported happened in the only patient in which mitomycin C was used, and so it may be correlated with its use. The anteriorization of the implant with trabeculodescemetic membrane rupture, registered at 6-month follow-up, did not show significant clinical repercussions or at the aqueous humor drainage, as noticed by the low IOP values measured. In contrast to other implants, such as Esnoper^®^, Esnoper-Clip^®^ is not sutured to sclera so it may predispose to implant dislocation, a complication that was never been documented in 5 years of experience of the authors with the first implant.

In the last few years, with new technological advances, filtering bleb structure has been studied [by confocal microscopy,^24,25^ ultrasound biomicroscopy (UBM)^20^ and AS-OCT^16-24^] in order to better understand the drainage pathways and its role on glaucoma surgery success. In this study, AS-OCT allowed to analyze the localization of the implant, the trabeculodescemetic membrane and hyporeflective spaces suggesting liquid content and so its drainage pathways, as already described in the literature.^16,17,19,20^ Esnoper-Clip® implant acts as a space maintainer and leads to the aqueous humor drainage to suprachoroidal, supraescleral and intraconjunctival spaces, as suggested by the images captured by AS-OCT. Therefore, this high-resolution imaging method may complement the clinical evaluation during the follow-up time.

In order to understand the relevance of this new implant in the clinical practice, further prospective, controlled, randomized and multicenter studies, with larger samples and longer follow-up, would be important. Moreover, studies should be done to compare DS with Esnoper-Clip^®^ with the use of other implants and with trabeculectomy with or without antimetabolites. Also, structure-function correlation studies, using AS-OCT, would be of value, as is already described for other implants.^17,19-21,23,24^

## CONCLUSION

Our first five DS with Esnoper-Clip® implant analysis suggest an effective and well-tolerated method to reduce IOP. AS-OCT is a noninvasive imaging technique that allows morphological analysis of the drainage mechanisms after glaucoma surgery.
